# Bioengineering of the Motor System

**DOI:** 10.3390/bioengineering12020199

**Published:** 2025-02-18

**Authors:** Carlo Albino Frigo

**Affiliations:** Department of Electronics, Information and Bioengineering, Politecnico di Milano, 20133 Milan, Italy; carlo.frigo@polimi.it

About fifty years ago, which seems very recent, new technologies for motion analysis were being developed, promising a more detailed and precise study of the human motor system. These technologies allowed the measurement of biomechanical variables and the quantification of phenomena that were previously difficult to assess through clinical observation. In the 1980s, the challenge from a bioengineering perspective was to make these systems as reliable and user-friendly as possible, enabling comprehensive movement analysis across various settings, including clinical environments.

Over the years, extensive research has been conducted to validate these technologies and develop application protocols, demonstrating their potential for research and clinical investigations. Today, well-established technologies such as stereophotogrammetric systems, dynamometric platforms, surface electromyography, and portable metabolimeters are commonly used in dedicated movement analysis laboratories. However, as technology has evolved, new demands and opportunities have emerged.

One of the most significant developments has been the increasing need for a more “ecological” approach to movement analysis, made possible by portable motion sensors. Research in this area has been highly active, leading to the widespread use of wearable systems based on miniaturized accelerometers, gyroscopes, and magnetometers, commonly known as Inertial Measurement Units (IMUs). Although IMUs have lower accuracy compared to complex stereophotogrammetric systems, they offer key advantages such as affordability, ease of use, and, most importantly, the ability to analyze individuals in natural settings such as gyms, sports fields, and workplaces. Additionally, the integration of these sensors into Virtual Reality systems has expanded their applications, enhancing our understanding of motor control mechanisms.

Another major advancement has been the improvement of computational power, which has facilitated the development of musculoskeletal modeling. Initially, these models provided representations of bones, joints, muscles, and ligaments, simulating the body’s degrees of freedom. The next step involved simulating movement by applying forces to the model, solving what is known as the forward dynamics problem. Once considered an unattainable challenge, this approach is now widely used by researchers to analyze the behavior of internal structures and assess the effects of modifying their mechanical properties.

These advances have led to a vast body of literature covering both technological developments and applications in diverse fields such as motor rehabilitation, neurology, orthopedics, sports science, ergonomics, and psychophysics. This Special Issue, “Bioengineering of the Motor System”, was conceived to collect research that exemplifies current interests in this field, and it has successfully fulfilled this objective.

An analysis of the twelve papers in this issue reveals that technological advancements now focus on developing algorithms to exploit data from existing systems [1,2] and incorporating sensorized devices [3,4]. Other studies explore applications involving Virtual Reality and exoskeletons [5,6,7]. Musculoskeletal modeling is represented in a study by [8] which highlights its potential for understanding internal anatomical structures during movement. Additional contributions focus on motor function recovery using electromyography [9], electrical stimulation [10], and specialized algorithms for identifying muscle synergies [11]. Finally, an intriguing study on human behavior examines psychological aspects using a motion capture system with a bioengineering approach [12].

These contributions illustrate how this Special Issue, entitled “Bioengineering of the Motor System”, has successfully assembled research that represents the state of the art and key areas of interest in this domain. Looking ahead, we can expect rapid advancements in methodologies based on current and emerging technologies. These developments will likely be directed toward clinical applications, significantly enhancing our understanding of the human motor system and driving progress in both research and practical implementations.
